# Vitamin D Receptor gene (VDR) transcripts in bone, cartilage, muscles and blood and microarray analysis of vitamin D responsive genes expression in paravertebral muscles of Juvenile and Adolescent Idiopathic Scoliosis patients

**DOI:** 10.1186/1471-2474-13-259

**Published:** 2012-12-23

**Authors:** Roman Nowak, Justyna Szota, Urszula Mazurek

**Affiliations:** 1 Orthopaedics Clinic Medical University of Silesia, Wojewódzki Szpital Specjalistyczny nr5 41-200 Sosnowiec, Pl. Medyków 1, Poland; 2 Department of Molecular Biology Medical University of Silesia, 41-100 Sosnowiec, ul.Narcyzów 1, Poland

**Keywords:** Idiopathic scoliosis, Vitamin D receptor gene, Spinal tissues QRT PCR analysis, Paravertebral muscle microarray analysis, Vitamin D responsive genes

## Abstract

**Background:**

VDR may be considered as a candidate gene potentially related to Idiopathic Scoliosis susceptibility and natural history. Transcriptional profile of VDR mRNA isoforms might be changed in the structural tissues of the scoliotic spine and potentially influence the expression of VDR responsive genes. The purpose of the study was to determine differences in mRNA abundance of VDR isoforms in bone, cartilage and paravertebral muscles between tissues from curve concavity and convexity, between JIS and AIS and to identify VDR responsive genes differentiating Juvenile and Adolescent Idiopathic Scoliosis in paravertebral muscles.

**Methods:**

In a group of 29 patients with JIS and AIS, specimens of bone, cartilage, paravertebral muscles were harvested at the both sides of the curve apex together with peripheral blood samples. Extracted total RNA served as a matrix for VDRs and VDRl mRNA quantification by QRT PCR. Subsequent microarray analysis of paravertebral muscular tissue samples was performed with HG U133A chips (Affymetrix). Quantitative data were compared by a nonparametric Mann Whitney U test. Microarray results were analyzed with GeneSpring 11GX application. Matrix plot of normalized log-intensities visualized the degree of differentiation between muscular tissue transcriptomes of JIS and AIS group. Fold Change Analysis with cutoff of Fold Change ≥2 identified differentially expressed VDR responsive genes in paravertebral muscles of JIS and AIS.

**Results:**

No significant differences in transcript abundance of VDR isoforms between tissues of the curve concavity and convexity were found. Statistically significant difference between JIS and AIS group in mRNA abundance of VDRl isoform was found in paravertebral muscles of curve concavity. Higher degree of muscular transcriptome differentiation between curve concavity and convexity was visualized in JIS group. In paravertebral muscles Tob2 and MED13 were selected as genes differentially expressed in JIS and AIS group.

**Conclusions:**

In Idiopathic Scolioses transcriptional activity and alternative splicing of VDR mRNA in osseous, cartilaginous, and paravertebral muscular tissues are tissue specific and equal on both sides of the curve. The number of mRNA copies of VDRl izoform in concave paravertebral muscles might be one of the factors differentiating JIS and AIS. In paravertebral muscles Tob2 and Med13 genes differentiate Adolescent and Juvenile type of Idiopathic Scoliosis.

## Background

Idiopathic scoliosis is the most common spinal deformity observed in humans. It is characterized by three dimensional changes of spinal shape observed as lateral curvature in frontal, thoracic lordosis in sagittal and axial rotation in horizontal plane. Besides the spine the deformity involves other bony structures including rib cage and pelvis. Despite many years of extensive experimental and clinical research the precise cause or causes leading to this crippling deformity affecting about 2-4% of otherwise healthy children were not ultimately defined. None of the many proposed theories explain entirely pathogenesis of the deformity or the causes of its progression that affects mainly girls during the periods of intensive growth. The enormous amount of data relating etiology of idiopathic scoliosis to structural elements of the spine, neuromuscular, proprioceptive, hormonal and biomechanical factors together with undisputable association with gender, growth and genetics indicates the multifactorial nature of this condition [1-3]. In accordance with the multifactorial nature of idiopathic scolioses is the concept of probably multiple genes predisposing to the deformity that could interplay with disease-modifier genes under the influence of diverse environmental and possibly epigenetic factors [4,5]. It seems generally accepted that girls with idiopathic scoliosis present a tendency to be taller and more slender than their peers [6,7]. Some studies indicate abnormal growth pattern and some abnormalities in body proportions [8]. Moreover remaining growth potential, skeletal maturity and menarchal status remain classic risk factors for the curve progression. Osteopenia has also been suggested as one of the risk factors for the progression of scoliotic deformity [9,10].

Vitamin D receptor gene VDR presents various genetic polymorphisms which potentially can affect its action within target tissues [11,12]. DNA polymorphisms in VDR gene appeared to be one of the most important determinants of height and menarchal age in girls [13,14]. The study of Xia and coworkers suggests an association between BsmI site polymorphism of VDR gene and abnormal growth pattern and low bone mass in girls with AIS [15]. Recently an association of VDR gene polymorphism with lumbar spine BMD in girls with idiopathic scoliosis was reported [16]. Although VDR may be considered as one of the candidate genes potentially related to idiopathic scoliosis susceptibility and natural history, as far little is known about the expression of this gene in the tissues of scoliotic patients. Multiple different transcript variants were found for the human VDR. The transcripts mostly use the same translation initiation codon and encode an identical 427 aminoacid-long VDR protein. A certain proportion of the VDR molecules are alternatively spliced. A product of alternative posttranscriptional splicing, the 50 aminoacid extended VDR1 can be found in most human cells [17]. This isoform coexists with classic isoform in humans. Functional differences between these two VDR isoforms can influence different transactivation capacity of certain promoters [18]. It is also possible that transcriptional profile of VDR isoforms might be changed in structural tissues of the scoliotic spine. These changes could potentially influence the expression of VDR responsive genes.

The onset of idiopathic scoliosis typically occurs in juvenile and adolescent period. Essential differences between the Juvenile and Adolescent Idiopathic Scoliosis include epidemiological data, natural history and response to the treatment [19-23]. It is also possible that some of these differences could be related to the alternative splicing of VDR exons. This study reports on VDR expression at the mRNA level in spinal tissues of the idiopathic scoliosis patients with different age of deformity onset pointing at differences in VDRl isoform transcript abundance and indicating Tob2 and Med13 as genes differentially expressed in paravertebral muscles of the curve concavity of Juvenile and Adolescent Idiopathic Scoliosis. The main aims of the study were:

Determination of differences in transcript abundance of VDR isoforms in osseous, cartilaginous and muscular tissues between curve concavity and convexity in Juvenile and Adolescent Idiopathic Scoliosis.

Determination of differences between Juvenile and Adolescent Idiopathic Scoliosis patients in mRNA abundance of VDR isoforms in bone, cartilage, paravertebral muscles and blood.

Identification of the VDR responsive genes in paravertebral muscular tissue that could differentiate Juvenile and Adolescent Idiopathic Scoliosis.

## Methods

**Figure 1:**
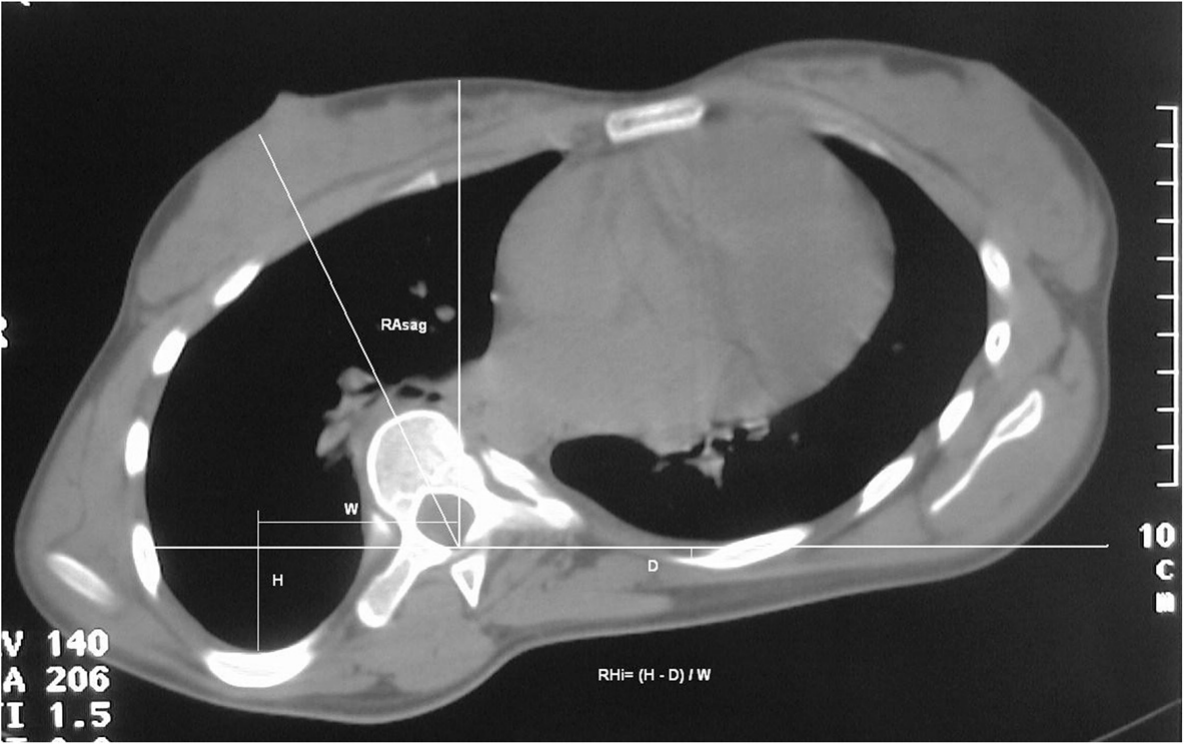
**Spinal and rib cage CT scan at the apex of the curve.** Measurement of the vertebral rotation angle about the longitudinal axis relative to the sagittal plane RAsag and Rib Hump index RHi defined as the ratio (H-D) / W.

Study design was approved by Bioethical Committee Board of Silesian Medical University. Informed, written consent was obtained from each patient participating in the study and if required from their parents. A group of twenty nine patients (24 females and 5 males) with a definite diagnosis of Idiopathic Scoliosis (other types of scoliosis of known origin excluded) had undergone posterior corrective surgery with segmental spinal instrumentation according to C-D method. Based on Lenke classification 6 curves were of type 1, 6 curves of type 2, 7 curves of type 3, 3 curves of type 4, 4 curves of type 5 and 3 of type 6 [24]. According to the age of scoliosis onset (diagnosis) 9 female patients were classified as a group A – Juvenile Idiopathic, presenting from age 3 to 10, and 20 patients (15 females and 5 males) as a group B-Adolescent Idiopathic Scoliosis, presenting from age 10 to 18 [25]. Preoperative radiological examinations were performed within one week before surgery and included posteroanterior (PA) and lateral radiographs of the spine taken in a standardized method in standing position, side bending posteroanterior radiographs made with the patient supine bending to the side of the curve convexity in order to correct the deformity in the frontal plane and CT scans of the thorax and spine performed at the curve apex. All radiographic measurements were performed by the same investigator with the same goniometer using Cobb’s method to assess the main curve severity in frontal and thoracic kyphosis in sagittal plane. The flexibility index (Fi) was calculated according to the formula: Fi = Cobb_standing_–Cobb_supine bending_/Cobb_standing_. The axial plane deformity was measured by spinal rotation angle relative to sagittal plane RAsag and rib hump index RHi as described by Aaro and Dahlborn, (Figure 1) [26]. During surgery bilateral facet removal was performed in the routine manner and bone and cartilage specimens from inferior articular spinal processes at the curve apex concavity and convexity were harvested. In the same time bilateral samples of paravertebral muscle tissue at the apical level and 10 ml of patient’s peripheral blood were collected. Every sample of bone, cartilage and muscular tissue as well as blood specimens were placed in separate sterile tubes, adequately identified and immediately snap frozen in liquid nitrogen and stored at -80°C until molecular analysis.

### Extraction of total RNA from osseous, cartilaginous, muscular and blood tissue samples

Tissue samples were homogenized with the use of Polytron® (Kinematyka AG). Total RNA was isolated from tissue samples with the use of TRIZOL® reagent (Invitrogen Life Technologies, California, USA) according to the manufacturer’s instructions. Extracts of total RNA were treated with DNAase I (Qiagen Gmbh, Hilden, Germany) and purified with the use of RNeasy Mini Spin Kolumn (Qiagen Gmbh, Hilden, Germany) in accordance with manufacturer’s protocol. The quality of RNA was estimated by electrophoresis on a 1% agarose gel stained with ethidium bromide. The RNA abundance was determined by absorbance at 260 nm using a Gene Quant II spectrophotometer (Pharmacia LKB Biochrom Ltd., Cambridge, UK). Total RNA served as a matrix for QRT PCR and microarray analysis.

*VDRs*, *VDRl*, *and endogenous controls* - *β actin and GAPDH mRNA quantification in osseous*, *cartilaginous*, *muscular and blood tissue samples by Quantitative Real Time Reverse Transcription Polymerase Chain Reaction*.

The quantitative analysis was carried out with the use of Sequence Detector ABI PRISM™ 7000 (Applied Biosystems, California, USA). The standard curve was appointed for standards of β actin (TaqMan® DNA Template Reagents Kit, Applied Biosystems, Foster, CA, USA).

**Figure 2:**
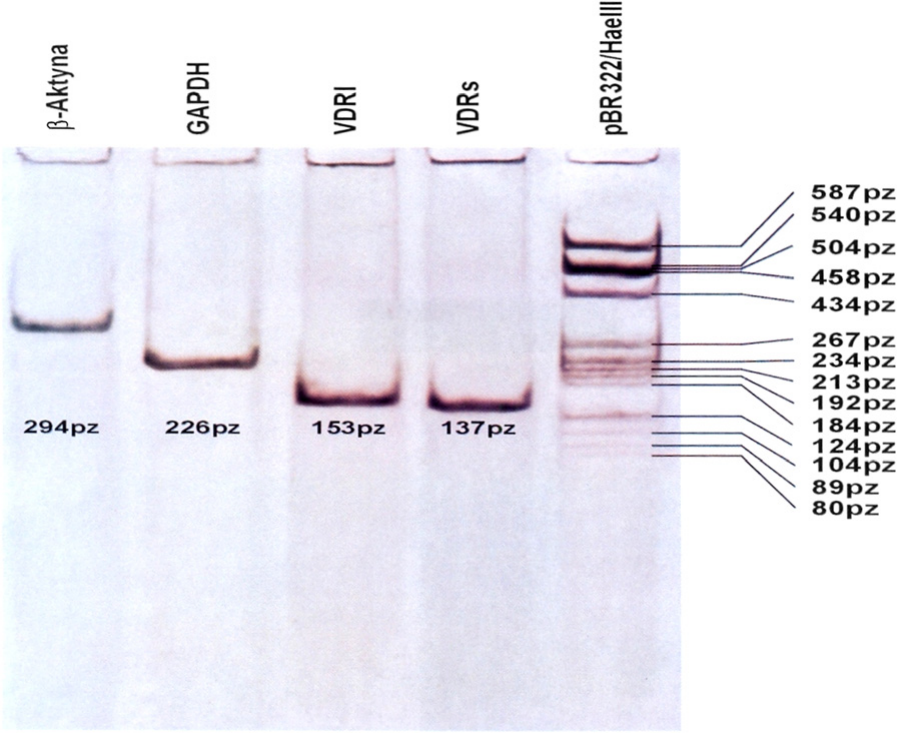
Results of electrophoresis of QRT-PCR products on 6% polyacrylamid gel.

**Figure 3:**
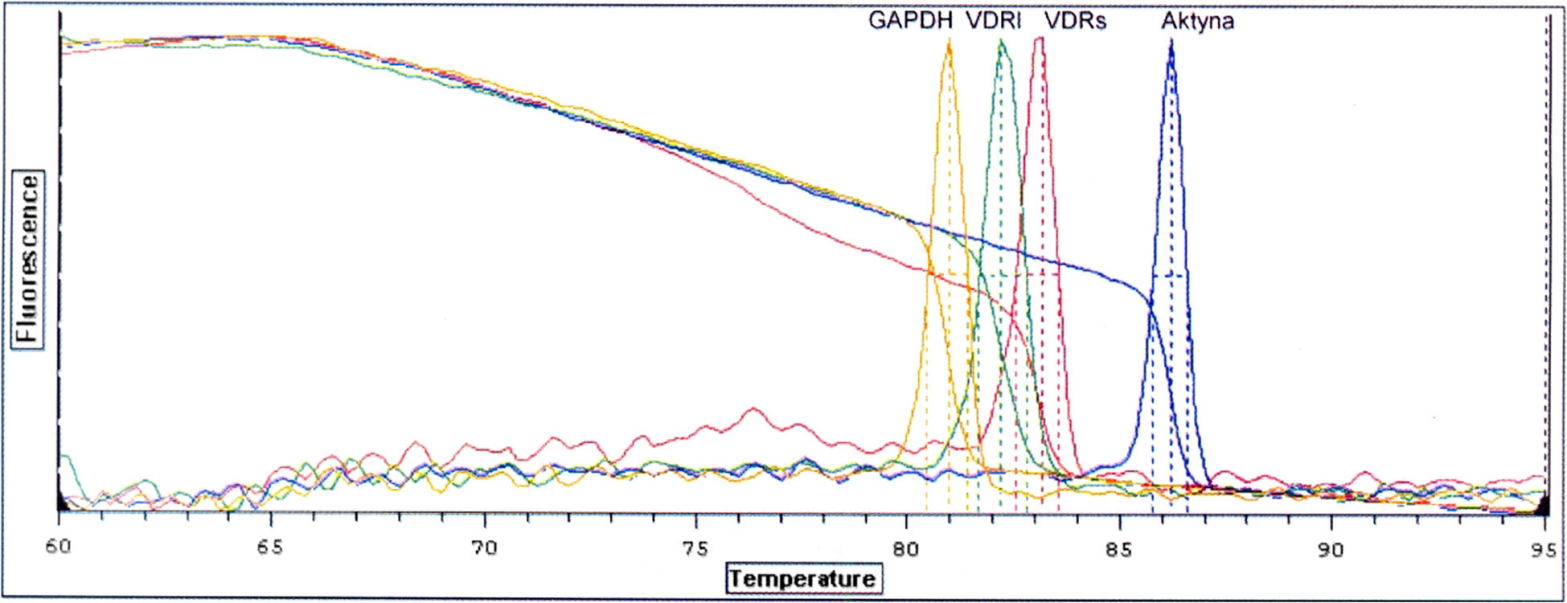
Dissociation curves indicating number of nucleic acids amplification products and T_m_ of amplimers: amplimer GAPDH (T_m_ = 80,1°C), amplimer VDR (T_m_ = 82,2°C), amplimer VDRs (T_m_ = 83,1°C), amplimer β-actin (T_m_ = 86,2°C).

Neither β actin nor GAPDH mRNA could serve as endogenous control as statistically significant differences (nonparametric U Mann Whitney test p<0,05) were found between number of copies of GAPDH and β actin between convex and concave side of the curve in bone and paravertebral muscles as well as between Juvenile and Adolescent Idiopathic Scoliosis in blood tissue samples. Thus VDRs and VDRl mRNA abundance in all studied tissue specimens was expressed as mRNA copy number per 1 μg of total RNA. The QRT-PCR reaction mixture of a total volume of 25 μl contained QuantiTect SYBR- Green RT-PCR bufor containing Tris–HCl (NH_4_)_2_SO_4_, 5mM MgCl_2,_ pH 8,7, dNTP mix fluorescent dye SYBR-Green I, and passive reference dye ROX mixed with 0,5 μl QuantiTect RT mix (Omniscript reverse transcriptase, Sensiscript reverse transcriptase) (QuantiTect SYBR-Green RT-PCR kit; Qiagen) forward and reverse primers each at a final concentration of 0,5 μM mRNA and total RNA 0,25 μg per reaction. Sequence for primers: mRNA for VDRl 5^′^TGAGGAATAAGAAAAGGAGCGATTGG3^′^ (forward primer) and 5^′^TGCCTCCATCCCTGAAGGAGAGG3^′^ (reverse primer) mRNA for VDRs, 5^′^CTTCCCTGCCTGACCCTGGAGACTTT3^′^ (forward primer), 5^′^GCTTCATGCTTCGCCTGAAGAAGCCTTTG3^′^ (reverse primer), mRNA for β actin 5^′^TCACCCACACTGTGCCCATCTACGA3^′^ (forward primer) 5^′^CAGCGGAACCGCTCATTGCCAATGG3^′^ (reverse primer), mRNA for GAPDH 5^′^GAAGGTGAAGGTCGGAGTC3^′^ (forward primer) 5^′^GAAGATGGTGATGGGATT3^′^ (reverse primer). Reverse transcription was carried out at 50°C for 30 min. After activation of the HotStar Taq DNA polymerase and deactivation of reverse transcriptases at 95°C for 15 min, subsequent PCR amplification consisted of denaturation at 94°C for 15 sec, annealing at 60°C for 30 sec and extension at 72°C for 30 sec (40 cycles). Final extension was carried out at 72°C for 10 min. QRT-PCR specificity was assessed by electrophoresis in 6% polyacrylamid gel and melting curves for aplimeres, (Figure 2 and Figure 3).

### Microarray analysis of paravertebral muscular tissue samples from convex and concave side of the curve apex with HG U133A chips (Affymetrix)

For both QRT-PCR and microarray analysis the same RNA samples acquired from muscular tissue from both sides of the curve were used. Muscular tissue samples preparation and microarray processing was performed according to Affymetrix Gene Expression Analysis Technical Manual. 6-8 μg purified total RNA was reverse transcribed with the use of SuperScript Choice System (Gibco BRL Life Technologies). First strand reaction mixture contained: 6–8 μg RNA, 0,5 μM primer T7 oligo(dT)_24,_ 1 × First Strand Buffer, 10mM DTT, 0,5 mM dNTPs, 200U SuperScript II RT. Second strand reaction mixture contained: 1 × Second Strand Buffer, 0,2 mM dNTPs, 40 U *E*. *coli* DNA I polymerase (Life Technologies), 10 U *E*. *coli* DNA ligase (TaKaRa), 2 U RNase H (TaKaRa), 10 U T4 DNA I polymerase (TaKaRa). dsDNA was purified with the use of Phase Lock Gel Light (Eppendorf). Biotynylated cRNA was synthesized with the use of BioArray HighYield RNA Transcript Labeling Kit (Enzo Life Sciences). Reaction mixture contained: 1 μg dsDNA, 1 × NTP mixture (NTP, Bio UTP, Bio CTP), 1 × HY buffer, 1 × dithiothreitol (DTT), 1 × RNase inhibitor, 1 × T7 polymerase RNA. cRNA was purified with the use of RNeasy Mini Kit (Qiagen). cRNA was fragmented by the use of Sample Cleanup Module (Qiagen). The reaction mixture contained: 16 μg of cRNA, 1 × buffer, ddH_2_O. Hybridization cocktail was prepared of 15 μg fragmented cRNA, 50 pM B2, eukaryotic controls (1,5 pM *bioB*, 5 pM *bioC*,25 pM *bioD*, 100 pM μl *cre*), 0,1 μg/ μl Herring Sperm DNA (Promega Corporation), 0,5 μg/μl BSA (Invitrogen Life Technologies), 1 × hybridization buffer (100 mM MES, 1M [Na+], 20 mM EDTA, 0.01% Tween 20) (Sigma Aldrich), 10% DMSO (Sigma Aldrich) (GeneChip® Expression 3^′^ Amplification Reagents Hybridization Control Kit). Hybridization on the microarray HG U133A was performed according to Affymetrix Gene Expression Analysis Technical Manual. Fluorescence intensity was measured with the use of Agilent Gene Array Scanner G2500A (Affymetrix).

### Statistical analysis

All calculations were performed with Statistica Version 8.0 software (StatSoft, Tulsa, Oklahoma, USA). The values were expressed as average, median, range and standard deviations. Quantitative data were compared by a nonparametric Mann Whitney U test and p<0.05 was considered statistically significant.

Statistical analysis of the microarray results was performed with the use of GeneSpring 11GX application (Agilent Technologies). Fluorescence intensity values of all 22 843 transcripts for 14 HG U133A chips were simultaneously normalized using RMA algorithm (Robust Multichip Average) [27]. Matrix plot of normalized log-intensities was used to visualize the degree of differentiation between muscular tissue transcriptomes of group A-JIS and group B-AIS harvested from curve concavity and convexity. The main purpose of the matrix plot is to get an overview of the correlation between conditions in the dataset, and detect conditions that separate the data into different groups. Higher intersample differences are displayed by more distant position of the spots from the regression line. Based on Affymetrix Net Aff database out of 22 843 transcripts 75 mRNA probes of VDR responsive genes that could be analyzed with HG U133A chips were selected [28]. To identify differentially expressed VDR responsive genes in muscular tissue samples in group A-Juvenile Idiopathic Scoliosis and group B-Adolescent Idiopathic Scoliosis Fold Change Analysis was performed with cutoff of Fold Change ≥2 assumed as significant. Fold change gives the absolute ratio of normalized intensities (no log scale) between the average intensities of the samples grouped. The results were visualized by scatter plots.

Microarray data of the experiment in MIAME-compliant format are publically available from the following address: http://www.ebi.ac.uk/arrayexpress/, the file ID is E-MTAB-980.

## Results

The results of the study were presented in three parts. In the first part patients of the group A-Juvenile Idiopathic Scoliosis and group B-Adolescent Idiopathic Scoliosis were characterized and compared based on seven clinical and radiological parameters. The second part was dedicated to the analysis of QRT PCR results. Differences in transcriptional profiles of VDRs and VDRl isoforms in osseous, cartilaginous, muscular and blood tissue were compared in relation to gender, side of the curve and age of the scoliosis onset. As statistically significant differences were observed only in the paravertebral muscles thus in the third part of the study transcriptome analysis of the paravertebral muscular tissue specimens with the use of HG U133A microarray chips (Affymetrix) was performed.

### Characteristics of the studied groups A-JIS and B-AIS

Both analyzed groups were statistically comparable according to the age at operation, skeletal age (Risser test), Cobb angle, thoracic kyphosis angle, axial rotation angle relative to the sagittal plane RAsag, rib hump index RHi and curve flexibility – Findex (Mann Whitney U test p>0,05), (Table 1).

**Table 1:** Characteristics and comparison of the studied groups

	**Group A Juvenile Idiopathic Scoliosis**					**Group B Adolescent Idiopathic Scoliosis**					**U-Mann Whitney**
**Parameter**	**Average**	**Median**	**Min**	**Max**	**St. dev**	**Average**	**Median**	**Min**	**Max**	**St. dev**	**p**
Age	17	15.9	14.8	24	2.92	16.8	15.6	13.7	25	3.03	0.54
Risser	4.2	4	4	5	0.44	3.6	4	1	5	1.19	0.21
Cobb	75.3	72	36	114	24.81	65.9	64.5	42	94	14.39	0.26
Kyphosis	42.8	48	12	70	17.28	32	28	12	59	14.12	0.09
RAsag	21.6	21	2.5	46	11.88	18.3	17.5	6	34	7.51	0.31
RHi	0.44	0.48	0.03	0.91	0.25	0.31	0.30	0	0.64	0.17	0.17
Findex	0.15	0.15	0	0.38	0.12	0.32	0.23	0	0.8	0.270	0.10

Radiological characteristics and statistical comparison of group A-juvenile idiopathic scoliosis (n=9) and group B- adolescent idiopathic scoliosis (n=20), p<0,05 was considered as a statistically significant (nonparametric Mann Whitney U test).

### Real Time QRT PCR results: VDRs and VDRl mRNA abundance in tissue samples

Group B-Adolescent Idiopathic Scoliosis included 20 patients (15 females and 5 males), thus the comparison of the VDRs and VDRl mRNA abundance in studied tissue samples between female (n=15) and male (n=5) population in this group was the first step of the QRT PCR data analysis. Subsequently VDRs and VDRl mRNA abundance in bone, cartilage and muscular tissue samples obtained from the concave side of the curve were compared with those from the curve convexity separately in both analyzed groups A-JIS and B-AIS. At the end group A was compared with the whole group B (5 males and 15 females) and with group B without males (n=15) regarding the transcript abundance of VDRs and VDRl in homonymous bone, cartilage, muscular and blood tissue samples.

Comparison of VDRs and VDRl mRNA abundance between female and male population in group B failed to show statistically significant differences in homonymous bone, cartilage and muscular tissue samples (p>0,05). However statistically significant difference between both sexes was found in VDRs mRNA abundance in blood samples, (p<0,05), (Table 2).

**Table 2:** VDRs and VDRl mRNA concentration in female and male population of group B-AIS

		**Group B (15 females)**				**Group B (5 males)**				**U-Mann Whitney**
**Sample**	**Isoform**	**Average**	**Median**	**Range**	**St. dev**	**Average**	**Median**	**Range**	**St. dev**	**p**
B1	VDRl	0	0	0		0	0	0		
	VDRs	240	150	11-746	300.5	7	7	2-13	7.3	0.09
B2	VDRl	0	0	0		0	0	0		
	VDRs	271	189	35-663	262	36	36	33-40	4.6	0.06
C1	VDRl	0	0	0		0	0	0		
	VDRs	1317	92	24-6193	2726.9	142	142	4-280	195.6	0.43
C2	VDRl	57	57	57		0	0	0		
	VDRs	3490	82	7-34119	10762	101	101	14-188	123.4	0.83
M1	VDRl	33	36	4-58	23.4	176	176	4-347	242.1	0.83
	VDRs	268	50	6-1649	561	290	375	8-487	250.4	0.95
M2	VDRl	49	43	3-132	43.6	77	77	37-117	56.8	0.79
	VDRs	641	17	1-4902	1612.5	372	165	44-906	466.8	0.50
Blood	VDRl	1215	1215	4-2427	1713.4	833	833	833		
	VDRs	695	695	30-1361	941.3	2621	2621	1800-3442	1160.6	0.04

Comparison of QRT-RTPCR results obtained from homonymous tissue samples (B1-bone from concavity, B2-bone from convexity, C1-cartilage from concavity, C2-cartilage from convexity, M1-muscles from concavity, M2-muscles from convexity, blood) between females (n=15) and males (n=5) in group B – Adolescent Idiopathic Scoliosis , p<0,05 was considered as a statistically significant (nonparametric Mann Whitney U test).

The next step of the data analysis was to compare mRNA abundance of VDRs and VDRl between tissues from concave and convex side of the scoliotic curve separately in group A and group B. As indicates Table 3, VDRs mRNA was present in all tissue samples of both studied groups as opposed to VDRl mRNA which was detected in both groups only in the muscular tissue and blood and in cartilage from the curve convexity in one case of the group B. However no statistically significant differences in the transcript abundance of VDRs and VDRl in bone, cartilage and paravertebral muscle tissue samples obtained from concave and convex side of the curve were found in neither of the studied groups (p>0,05), (Table 3).

**Table 3:** Comparison of tissues from concave and convex side of the curve

**Group**	**Isoform**	**Sample**	**Average**	**Median**	**Range**	**St. dev.**	**p**
A	VDRl	B1	0	0	0		
		B2	0	0	0		
	VDRs	B1	821	131	3 - 3018	1467	1.0
		B2	734	643	70 -1579	780	
B	VDRl	B1	0	0	0		
		B2	0	0	0		
	VDRs	B1	174	30	2-746	270.3	0.28
		B2	204	66	33-663	242.6	
A	VDRl	C1	0	0	0		
		C2	0	0	0		
	VDRs	C1	43754	193	7-252723	102440.5	0.72
		C2	6901	98	89-34119	15213	
B	VDRl	C1	0	0	0		
		C2	57	57	57		
	VDRs	C1	981	92	4-6193	2300.6	0.66
		C2	83	66	14-188	61.5	
A	VDRl	M1	1493	218	56-5478	2661.3	0.64
		M2	348	348	7-688	481.8	
	VDRs	M1	3526	377	35-17817	6599.6	0.75
		M2	1204203250.5	185	22-8429421053	3186021578.8	
B	VDRl	M1	69	36	4-347	114.5	0.75
		M2	56	43	3-132	44.6	
	VDRs	M1	274	61	6-1649	482.7	0.50
		M2	574	38	1-4902	1394,8	

Comparison of QRT-PCR results between tissue samples obtained from concave and convex side of the scoliotic curve in group A – Juvenile Idiopathic Scoliosis (n=9) and group B-Adolescent Idiopathic Scoliosis (n=20). B1, C1, M1 adequately bone, articular cartilage and paravertebral tissue samples from concave side of the curve. B2, C2, M2 adequately bone, articular cartilage and paravertebral tissue samples from convex side of the curve, p<0,05 was considered as a statistically significant (nonparametric Mann Whitney U test).

Finally group A – JIS was compared with whole group B –AIS (5 males and 15 females) and with group B without male patients (n=15 females only) according to mRNA abundance of VDRs and VDRl in homonymous bone, cartilage, muscular and blood tissue samples.

The only statistically significant difference found between the two studied groups was in the number of copies of VDRl mRNA in paravertebral muscles acquired from curve concavity (M1), (p=0,04). This difference was further accentuated when only female population of both groups was considered, (p=0,03), (Table 4).

**Table 4:** Comparison of juvenile and adolescent idiopathic scoliosis tissue samples

	**Group A vs B (girls and boys)**							**Group A vs B (girls)**						
	**B1**	**B2**	**C1**	**C2**	**M1**	**M2**	**Blood**	**B1**	**B2**	**C1**	**C2**	**M1**	**M2**	**Blood**
	**p**	**p**	**p**	**p**	**p**	**p**	**p**	**p**	**p**	**p**	**p**	**p**	**p**	**p**
VDRl					0.0415	0.7940	0.2482					0.0330	0.7389	0.44
VDRs	0.5708	0.1306	0.4751	0.5698	0.1130	0.1282	0.3272	1.0000	0.3272	0.7150	0.6015	0.1052	0.0807	0.70

Comparison of QRT-RTPCR results in homonymous bone, articular cartilage, paravertebral muscle tissues and blood samples between group A – Juvenile Idiopathic Scoliosis and group B- Adolescent Idiopathic Scoliosis. Comparison of group A with group B without male patients on the right side of the table. B1, C1, M1 adequately bone, articular cartilage and paravertebral tissue samples from concave side of the curve. B2, C2, M2 adequately bone, articular cartilage and paravertebral tissue samples from convex side of the curve, p<0,05 was considered as a statistically significant (nonparametric Mann Whitney U test).

Data concerning transcript abundance of both VDR isoforms in group A-Juvenile Idiopathic Scoliosis and group B-Adolescent Idiopathic Scoliosis in blood tissue are presented in Table 5.

**Table 5:** Blood tissue concentration of VDRl and VDRs mRNA in group A-juvenile idiopathic scoliosis and group B-adolescent idiopathic scoliosis

**Group**	**Isoform**	**Average**	**Median**	**Range**	**St. dev.**
A	VDRl	2368	2368	987 - 3749	1953.6
	VDRs	751	654	407-1371	401.7
B	VDRl	1088	833	4-2427	1231.5
	VRDs	1658	1581	30-3442	1407.4

### Microarray analysis

**Figure 4:**
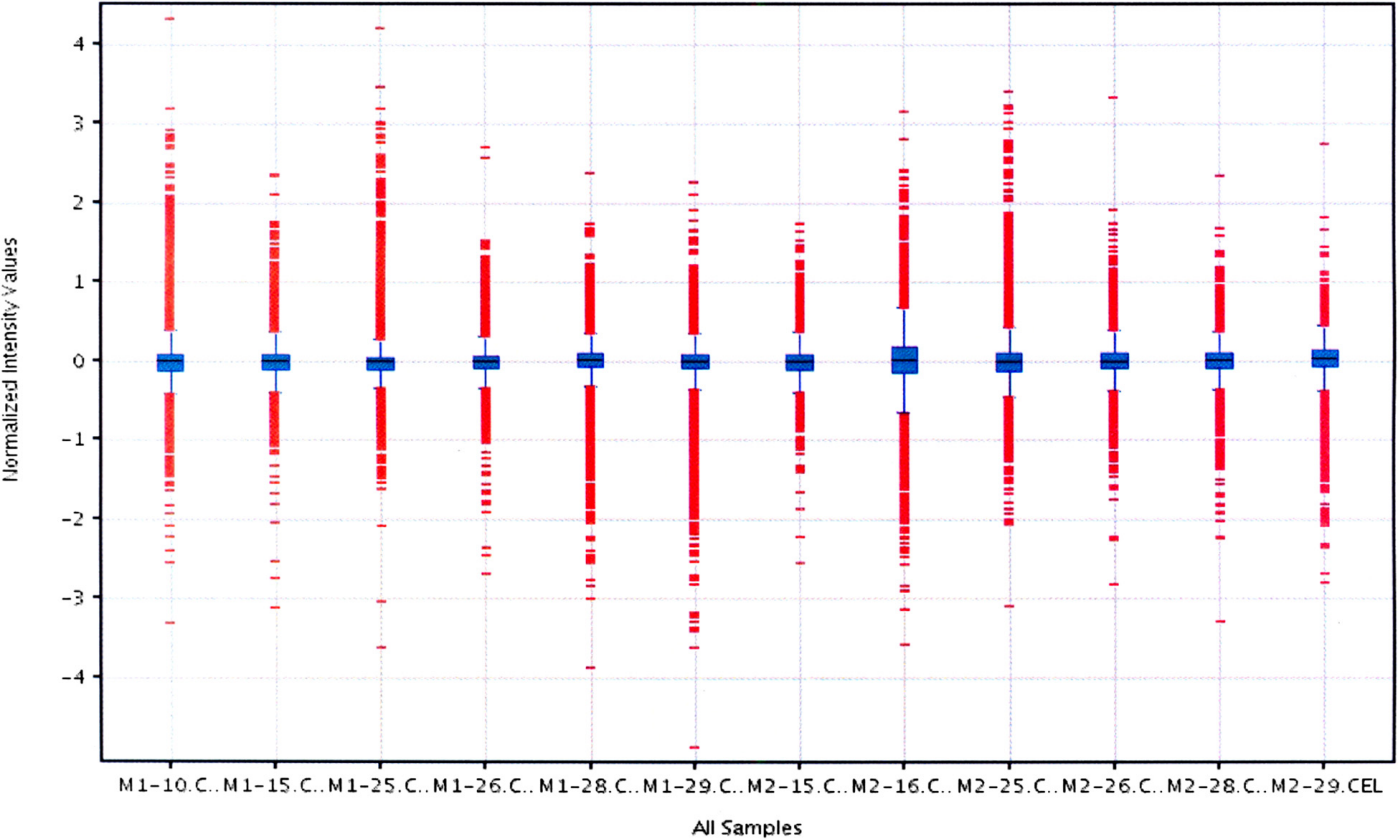
**Comparison of fluorescence intensity values of 12 paravertebral muscle HGU 133 A (Affymetrix) oligonucleotide microarray chips (M1, M2 – adequately muscular tissue from curve concavity and convexity).** Every box-and-whisker plot represents 22283 mRNA probes fluorescence intensity values normalized with RMA algorithm. All signals based to median (black horizontal lines), height of the rectangle (blue) determines the value of interquartile range; outliers marked in red.

**Figure 5:**
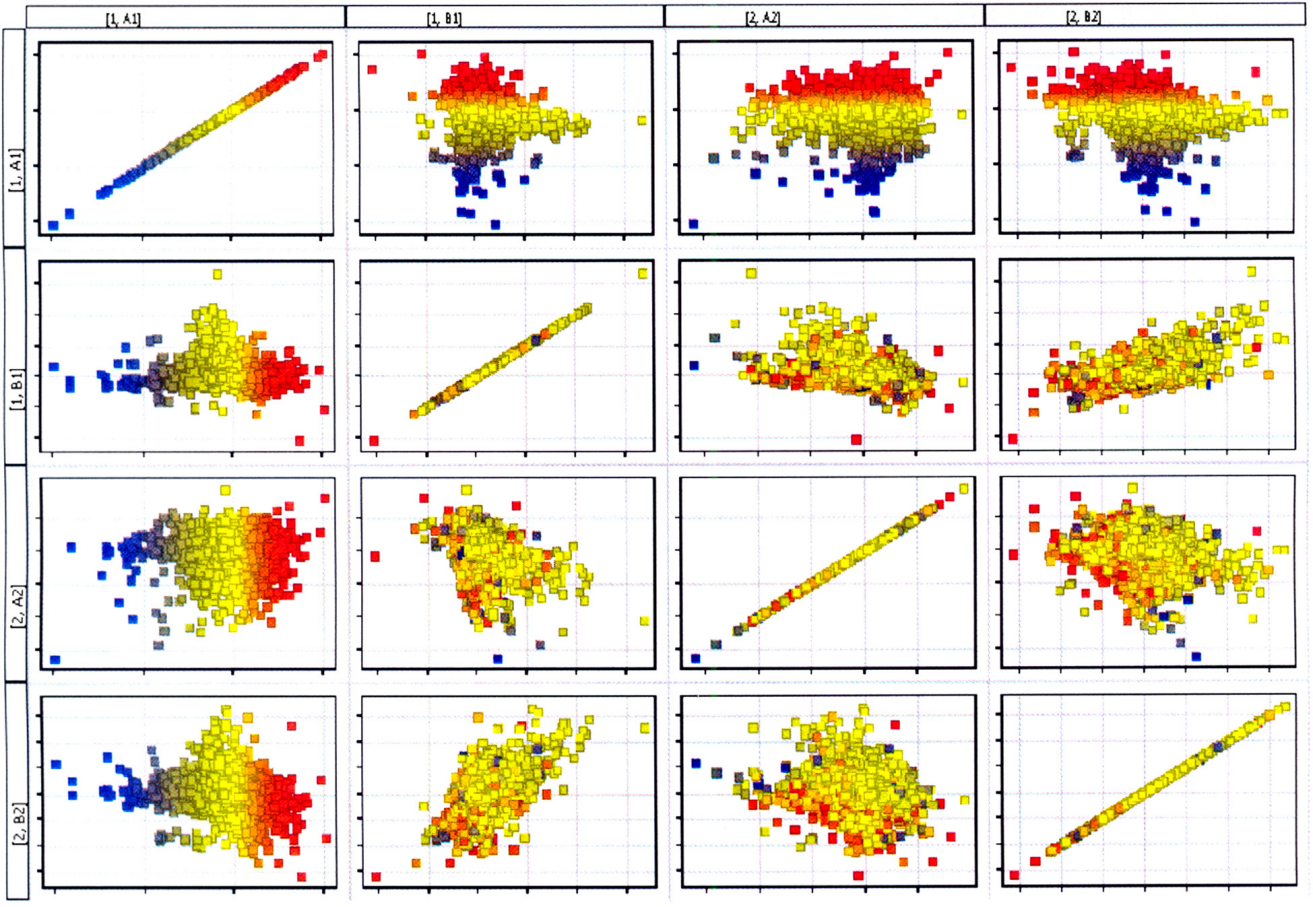
**Matrix plot illustrating the degree of differentiation between the transcriptomes of muscular tissue in dependence of the side of the curve and the age of scoliosis onset.** Red spots-up regulated genes, blue spots-down regulated genes. A1, A2- adequately muscular tissue samples from curve concavity and convexity in group A-Juvenile Idiopathic Scoliosis; B1, B2- adequately muscular tissue samples from curve concavity and convexity in group B-Adolescent Idiopathic Scoliosis.

**Figure 6:**
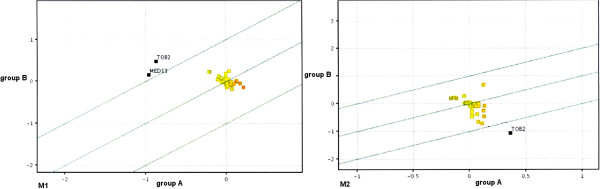
**Scatter plot comparing normalized intensities of 75 mRNA probes of VDR responsive genes between group A –JIS and B-AIS in muscular tissue specimens from the concave (M1) and convex side of the curve (M2).** Each rectangle represents the normalized expression of an individual gene within both mRNA populations. Axes of the scatter plot represent the log scale of the normalized fluorescence intensity value in group A and B. The middle dashed line indicates values that represent a ratio of 1 (similar expression in group A and B). The outer lines represent a ratio of 2,0 (upper line; 2-fold greater expression in group B compared with group A) and 0,5 (lower line; 2-fold greater expression in group A compared with group B).

Fourteen RNA samples, 7 acquired from paravertebral muscular tissue from curve concavity (M1) and 7 from curve convexity (M2) of 4 patients from group A – Juvenile Idiopathic Scoliosis and 3 patients from group B- Adolescent Idiopathic Scoliosis were subjected for microarray experiment. Preliminary qualitative analysis permitted to include 12 HGU 133A oligonucleotide chips for further analyses, (Figure 4). In order to assess the differences between the muscular tissue transcriptomes harvested from curve concavity and convexity in group A-JIS and group B-AIS matrix plot analysis of the mRNA expression data was performed, (Figure 5). This analysis permitted to visualize higher degree of differentiation between muscular tissue transcriptomes harvested from curve concavity and convexity in group A-Juvenile Idiopathic Scoliosis (A1 versus A2) compared to group B-Adolescent Idiopathic Scoliosis where these differences were less pronounced (B1 versus B2). Generated matrix plot indicated also higher differences between group A and B in gene expression at mRNA level in the specimens from the curve concavity (A1 versus B1). Much less differentiation could be observed between the transcriptomes from the curve convexity (A2 versus B2). The results of the QRT PCR in this study indicated statistically significant difference between group A-JIS and B-AIS in mRNA abundance of VDRl isoform in muscular tissue from curve concavity. An assumption was made that such a change in VDRl mRNA expression profile could be reflected by changes of the expression profile of the VDR responsive genes. In order to identify VDR regulated genes differentially expressed in paravertebral muscular tissue from both sides of the curve in group A-JIS and group B-AIS a group of 75 mRNA probes of VDR responsive genes was selected out of 22 843 transcripts. Selection was made based on Affymetrix Net Aff database [28]. Subsequently a Fold Change Analysis was performed in search for VDR responsive genes differentiating Juvenile and Adolescent Idiopathic Scoliosis in paravertebral muscular tissue samples. The cut off was set at Fold Change ≥ 2. The results of the analysis are presented as scatter plots, (Figure 6). In the muscular tissue samples harvested form curve concavity only two out of the 75 VDRs responsive genes appeared to be differentially expressed in group A-JIS and B-AIS: Tob2 and MED13. Both genes were up regulated in the population of group B- Adolescent Idiopathic Scoliosis. Tob2 gene was also differentially expressed at the curve convexity, but its expression was up regulated in the population of group A-Juvenile Idiopathic Scoliosis.

## Discussion

VDR expression with variable intensity has been confirmed in virtually every human tissue [18]. A broad set of microarray studies performed to unravel the molecular pathways involved in the biological action of ligand activated vitamin D receptor VDR indicate that VDR regulates directly or indirectly a very large number of genes (0,5-8% of total genome) and appears to be involved in a variety of cellular functions including growth regulation, DNA repair, differentiation, apoptosis, membrane transport, metabolism, cell adhesion, and oxidative stress [18]. Besides the direct regulation of gene transcription VDR exerts its function through nongenomic actions. This activity includes a number of transitient changes in transmembrane transport of ions (such as calcium and chloride) or intracellular signaling pathways (such as changes in cAMP, protein kinase A, protein kinase C, phospholipase C, phosphatidylinositol-3 kinase, and MAPK activities). Nongenomic actions are transmitted by receptors localized in the cellular membrane [18]. Different transcript variants of VDR gene encode either 427 aminoacid long classic isoform VDRs or alternatively spliced 50 aminoacid extended isoform VDRl [17,18]. Low levels of endogenous VDRl protein have been detected in osteoblasts, colon cancer, and kidney cell lines. VDRl protein has reduced transcriptional activity compared with classical VDRs [29]. Whether the levels of expression of these isoforms have implications for altered activities such as transactivation function or subcellular localization of the receptor protein *in vivo* remains a mystery. Furthermore, these variants, by their concentration level, tissue specificity, subcellular localization and functional activity, may yield potential targets for pharmaceutical intervention. The results of our study indicate that coexistence of VDRl and VDRs at transcriptional level might be tissue specific. Presence of classic VDRs isoform mRNA was confirmed in all studied tissues. However VDRl mRNA was absent in bone tissue specimens in both analyzed groups regardless of the side of the curve, Table 3. Also in the cartilage VDRl mRNA was present only in one patient of the group B-Adolescent Idiopathic Scoliosis in the specimen obtained at the curve convexity (C2). Presence of VDRl mRNA confirmed in paravertebral muscles from both sides of the curve and in blood samples of both analyzed groups might further support the thesis of the tissue specific role played by this isoform. The question if the observed absence of VDRl mRNA in bone tissue is inherent to Idiopathic Scoliosis could be addressed but small number of the analyzed specimens and lack of the control group in this study precludes concluding answer. In the literature we found no data concerning analysis of the transcriptional profile of the VDR gene in the tissues of the scoliotic patients.

Idiopathic Scoliosis is one of the clinical disorders with evident sexual dimorphism expressed mainly by differences in epidemiology and risk of progression [30]. Female preponderance is especially evident in progressive cases necessitating operative treatment. The female/male ratio for curves greater than 30^0^ approaches 10:1 [23]. The fact that bone, cartilage and skeletal muscles in humans exhibit sexual dimorphism is well known [31-33]. The differences between the sexes in skeletal dimensions and shape, biomechanical responses, mineral mass, bone turnover and trabecular microstructure are obvious [31]. As we learnt from animal models VDR deficiency causes sexually dimorphic changes in bone phenotype. The skeletal phenotype of VDR null mice shows that the absence of VDR reduces bone tissue mineralization, and this effect is more significant in males [18]. The exact mechanisms responsible for these differences are not clear. One of the possibilities is that there are differences in vitamin D receptor expression in female and male cells. In the literature we found no data concerning the quantitative analysis of sex related differences in VDR isoforms mRNA abundance in humans. Results of the QRT PCR of this study indicate that at the mRNA level in osseous, cartilaginous and muscular tissues of the spine transcript abundance of both VDR isoforms in female and male population is comparable. However significantly higher mRNA abundance of VDRs isoform was found in blood samples of the male population (p<0,05). Further investigations should be performed to answer the question whether the differences in mRNA abundance of VDRs isoform between female and male population are tissue specific. Lack of statistically significant differences in osseous, cartilaginous and muscular tissues of scoliotic patients might suggest that at least at the spinal level transcriptional profile of the mRNA isoforms of the vitamin D receptor is not sex-related.

Although there is still no generally accepted theory for the etiopathogenesis of idiopathic scoliosis most of the researchers agree with the multifactorial nature of this disorder [2,3,34,35]. Multifactorial nature of idiopathic scoliosis is well illustrated by the genetic model proposed by Cheng et al. with a set of genes responsible for the initiation and another set involved in the curve progression [4]. These genes could act separately or interact and probably be influenced by diverse environmental and possibly epigenetic factors [4,5,34]. In this context IS could be a systemic molecular disorder or disorders reflected at least in part by local processes affecting musculoskeletal structures of the spine during periods of intensive growth and development. Once initiated a few of the curves progress and become severe enough to necessitate intensive treatment. In these curves progression may occur because of strong spinal asymmetries inducing eccentric loading of the spine and in consequence asymmetric growth of pedicles, vertebral bodies and arches in accordance with Heuter-Volkmann effect [36-40]. Bone microarchitecture structural changes of spinal facets harvested at the curve apex of the scoliotic patients consistent with remodeling pattern of eccentrically loaded spine were confirmed by electron microscopy [41]. Whether these changes are solely secondary or related to an underlying pathological bone condition remains unclear. Lowered BMD is one of the potential factors that may be responsible for decreased resistance to mechanical loading [42]. Decreased bone mineral density was found in 27-68% of children with idiopathic scoliosis [42-48]. Histomorphometric studies of bone biopsies harvested from patients with idiopathic scoliosis revealed decreased differentiation of mesenchymal stem cells into osteoblasts and abnormal osteoclasts activity [45,48]. After binding to its nuclear receptor VDR, the complex 1,25-Dihydroxyvitamin D3 – VDR can influence bone metabolism indirectly by promoting intestinal absorption of calcium phosphate and directly by acting on osteoblasts and osteoclasts [16]. Also in chondrocytes during skeletal growth VDR expression affects trabecular bone mass [18]. In consequence VDR gene is one of the major candidate genes responsible for osteoporosis and fracture risk [49,50]. Recently an association was found between VDR polymorphism and lumbar spine BMD in Adolescent Idiopathic Scoliosis [16].

The essential role of paravertebral muscles in stabilizing the spine and controlling its dynamic functions is not to be questioned [51,52]. Thus it is not astonishing that a primary muscle disorder has been postulated as a possible etiology of idiopathic scoliosis. Paravertebral muscle abnormalities noted in idiopathic scoliosis patients include decreased number of slow twitch type I fibers on the concavity accompanied by a higher proportion of fast twitch type II fibers on both sides of the curve compared with controls [53]. Molecular and cellular details of the mode of action of vitamin D –VDR complex on striated muscles are scarce. The information gained from VDR null mice model support a direct role of vitamin D and VDR in metabolic processes and transcriptional regulation of skeletal muscles [18]. Vitamin D and VDR exert their action on muscles through both genomic and nongenomic mechanisms affecting transcription of calcium related proteins and total body calcium levels [18]. Any perturbation in calcium homeostasis may influence the contractile and relaxation properties of skeletal muscles. VDR expression in muscular tissue is especially marked during the early myoblast and myotube stages of development. In fully mature muscles expression is much lower. However muscle fibers of type I and type II of all striated muscles are significantly smaller in VDR null mice which suggest the role of VDR in the late muscle development [18]. A significant relationship was also observed between serum vitamin D levels and IIa fiber area in an experimental murine study indicating possible influence of VDR activity on distribution of fiber type [54]. The contractile proteins of platelets resemble those of skeletal muscle and calmodulin is an important mediator of calcium-induced contractility. Previous studies have shown that an increased calmodulin concentration in platelets is associated with progression of AIS [55,56]. Higher concentration of calmodulin in paravertebral muscles at curve convexity were demonstrated in patients undergoing surgery for AIS [57]. One of the possible explanations of the asymmetrical calmodulin distribution might be differences in VDR activity between concave and convex side of the curve, as binding of 1,25 dihydroxyvitamin D to its receptor activates a genomic pathway leading to an increase in the synthesis of calmodulin in proliferating myoblasts [58]. Knowledge of the potential role of posttranscriptional variants of VDR in bone, cartilage and muscular tissues is lacking. One may presume that an asymmetry in the tissue distribution of both VDR isoforms between concave and convex side of the curve could be of importance in the IS pathogenesis or be implicated in curve progression. However the results of QRT PCR of this study didn’t show significant differences in transcript abundance of VDRs and VDRl between concave and convex side of the curve in bone, cartilage and paravertebral muscle tissue in neither of the studied groups (p>0,05).

### Juvenile versus adolescent type of idiopathic scoliosis

Idiopathic Scoliosis is usually diagnosed in juvenile and adolescent period [59]. The age of scoliosis onset in large extent determines its epidemiology, natural course and response to the treatment [19,21-23]. Juvenile curves represent 12-21% of all scolioses with unknown etiology [19,21,22,60]. Sex related differences in prevalence place juvenile idiopathic scoliosis between infantile and adolescent type. In younger population between 4 and 6 year old female/male ratio is equal. Later (7–9 year of age) girls start to predominate with the ratio 4,5:1 and about the age of ten female/male proportion of 8:1 resemble ratio observed in adolescent type of scoliosis [60]. Curves morphology is similar in both types of scolioses with predomination of right primary thoracic and double primary thoracolumbar [22,60]. The most important factor discriminating juvenile from adolescent type is the risk of deformity progression. Because of severe progression 27% to 80% of juvenile curves necessitate operative treatment, whereas in adolescent type of idiopathic scoliosis risk of progression is much lower and only 0,1% of patients are subjected to operation [19,21-23,61,62]. The cause or causes of different age of scoliosis onset and related differences in natural history rest to be elucidated. Results of heritability study of 69 extended Utah families with a history of AIS indicate that onset of AIS is inherited separate from curve pattern and severity [63]. It seems also that genetic markers used for progression of adolescent type of idiopathic scoliosis do not apply to early onset idiopathic scoliosis below 9 years of age [64]. Vitamin D receptor gene VDR may be considered as one of the candidate genes potentially related to idiopathic scoliosis susceptibility and natural history. One of the aims of this study was to evaluate the differences between juvenile and adolescent type of idiopathic scoliosis in tissue transcriptional abundance of VDR gene isoforms. Specimens of bone, cartilage, muscular and blood tissues obtained from patients with juvenile and adolescent type of idiopathic scoliosis were submitted to QRT PCR analysis. Analysis of the obtained results indicates that both groups differ significantly in the mRNA abundance of VDRl isoform in paravertebral muscles of the curve concavity. The exact meaning of this finding is unknown although one may presume that difference in transcript abundance of VDRl isoform in muscular tissue of curve concavity might be of importance for some of the differences between juvenile and adolescent form of idiopathic scoliosis including different potential of progression. However lack of differences in mRNA abundance of both VDR isoforms between muscular tissue from curve concavity and convexity in both analyzed groups suggests precariousness in drawing definite conclusions concerning the role of the posttranscriptional modifications of VDR gene in curve progression. Another important limitation of this study that should be addressed is the fact that all of the tissue samples were harvested from patients with severe curves, long after the deformity onset. It is certainly a problem when patients with severe idiopathic scoliosis are selected for the experimental group because such patients represent the extreme cases and they are usually much older than when the curve started to develop [35]. So it should be pointed out that molecular differences observed in this study between Juvenile and Adolescent Idiopathic Scoliosis may as well reflect different time of curve evolution from the deformity onset to the time of the operative treatment.

### Paravertebral muscles microarray analysis

The results of the statistical analysis of the QRT PCR results obtained from different tissues of the studied groups unraveled statistically significant difference between patients with Juvenile and Adolescent Idiopathic Scoliosis solely in paravertebral muscles from the curve concavity. Consequently paravertebral muscle tissue specimens obtained from curve concavity and curve convexity of both analyzed groups were submitted for microarray analysis of 22 843 transcripts. Matrix plot analysis of the transcriptomes visualized some differences in gene expression between the two groups of patients with different idiopathic scoliosis onset. Primarily in group A with Juvenile Idiopathic Scoliosis higher degree of differentiation between transcriptomes from curve concavity and convexity with more up and down regulated genes could be noted. This observation could be related with the higher progression potential of the curves with earlier onset. Potential differences in the etiopathogenesis of both groups of idiopathic scolioses with paravertebral muscular tissue involvement in Juvenile Idiopathic Scoliosis might be another possible explanation. Matrix plot analysis permitted also to localize higher differentiation of the transcriptomes between Juvenile and Adolescent Idiopathic Scoliosis group at the curve concavity. This observation might suggest paravertebral muscles of curve concavity as a potential target of future molecular research. QRT PCR results showed that in the muscular tissue samples from the concave side of the curve mRNA abundance of VDRl isoform was significantly higher in Juvenile than in Adolescent Idiopathic Scoliosis group. Although the exact role of the VDRl isoform in human physiology rest to be elucidated one may presume that changes of the tissue transcript abundance of this isoform might be reflected by the changes in the expression profile of the VDR responsive genes. Consequently the next step of the microarray data analysis was directed to identify VDR regulated genes differentially expressed in Juvenile and Adolescent Idiopathic Scoliosis group in muscular tissue samples from both sides of the curve. Fold change analysis of the results permitted to identify Tob2 and MED13 as VDR responsive genes differentially expressed in Juvenile and Adolescent Scoliosis group in muscular tissue samples of curve concavity. Both genes were up regulated in Adolescent Scoliosis group. Interestingly Tob2 was also differentially expressed at the curve convexity but appeared to be up regulated in Juvenile Idiopathic Scoliosis. Tob2 is one of the members of Tob /BTG or APRO family of antiproliferative proteins that modulate cell cycle progression from phase G_0_/G_1_ to S and play diverse roles in development and in other biological processes including cell differentiation, and cell movements during embryogenesis [65]. Because of the lack of DNA-binding domain Tob proteins act as coactivators or corepressors in conjunction with various transcription factors [66]. Tob genes seem to play role in early and later stages of embryogenic development. In amphibian and fish embryos Tob proteins play role in dorsoventral patterning through inhibition of transcriptional stimulation by β-catenin, essential factor for the dorsal development. β-catenin may be one of the major targets of Tob proteins for exerting their antiproliferative effect [65]. During segmentation expression of Tob genes was confirmed in somites, which ultimately give rise to axial skeleton, skeletal muscle and dermis [65]. It appeared also that Tob1gene was differentially expressed throughout skeletal muscle development and contributed to phenotypic differences in muscle in experimental animals [66]. Tob can associate with the Smads transcription complex and affect Smad-mediated gene expression [67]. Phosphorylation of Tob1 by ERK1 and ERK2 negatively regulates the antiprolipherative activity of Tob1 [65]. Both Tob1 and Tob2 also interact with human Caf1 and form transcriptional complexes that activate or suppress target gene transcription [65]. Tob genes play also important role in bone formation and resorption. Tob1 controls bone formation by suppressing BMP signaling and by inhibiting sex hormone signaling in osteoblastic cells [65]. Tob2 decreases osteoclasts differentiation and regulates RANKL expression in stromal cells. Probably in regulating RANKL expression Tob2 interacts with VDR [67]. It seems that Tob1 and Tob2 may interfere in bone formation as Tob1 deficient mice present with osteopetrotic phenotype while Tob2 deficient animals are osteoporotic [67]. How Tob2 interacts with VDR in skeletal muscles remains a mystery. The exact biological role played by VDR posttranscriptional modifications is also unknown. Our results indicate that significantly lower concentration of VDRl mRNA found in Adolescent Idiopathic scoliosis group compared with their juvenile peers coincide, at least at the transcriptional level, with the up regulation of Tob2 gene in paravertebral muscles of the curve concavity. In the same time Tob2 was down regulated in the AIS group compared to JIS in the muscular tissue of the curve convexity. Tob proteins have ability to function as transcriptional regulators and can modulate growth in a manner dependent on the cell type and molecular context. Their interaction with Smads link them to the TGFβ family mediated signaling and regulation of transcription. Smad 2 and 3 are the transcription factors downstream of TGFβ and can play crucial role in activation of atrophy program in skeletal muscles [68]. Tob is a negative regulator of Smads [69]. Inhibition of Smads in skeletal muscles promotes myofiber hypertrophy [68].

MED 13 appeared to be another VDR responsive gene differentiating Juvenile and Adolescent Idiopathic Scoliosis group in the muscular tissue specimens from the curve concavity. MED13 protein is a component of a conserved multisubunit complex of Mediator (MED). Mediator MED represents a major subassembly of the preinitiation complex that plays numerous roles in controlling its function and is required for expression of RNA polymerase II dependent genes [70,71]. Mammalian Mediator complex was found to exist in multiple forms composed of different subunits [72]. Together with MED12, cyclin-dependent kinase (CDK8) and cyclin C (CycC), MED13 form a separable Mediator subcomplex known as “CDK8 module”. MED13 seems to play a critical role for linking the CDK8 module to the core of Mediator. This association may lead to repression of activated transcription and thus modulate and control transcript levels [71,72]. MED12 and MED13 appeared also to be required for transcriptional activation by other transcription factors like Nanog, members of GATA and RUNX families and yeast Pdr3 [73]. Mediator-like complexes have been purified in association with the liganded vitamin D receptor VDR and designated DRIP (Vitamin D receptor interacting protein complex) [74]. MED13 was identified as one of the DRIP/Mediator subunits together with MED12, MED1, MED14, MED23, MED24, MED16, MED17 and MED6 [75]. Although interaction of mediator core subunit MED1 appears to be critical for optimal recruitment of the Mediator to nuclear receptor regulated genes, it appears that nuclear receptors may target other mediator subunits in addition to MED1 and different Mediator subunits can play dominant roles in regulation of different genes by the same nuclear receptor [73]. Up to date the exact role of the Mediator subunit MED13 in VDR activity is unknown. Possibly multiple molecular mechanisms are engaged in the pathogenesis and evolution of idiopathic scolioses. The results of genetic association studies of the last decade permitted to point out some of the genes potentially involved in the occurrence of Adolescent Idiopathic Scoliosis. The list include genes of estrogen receptors ERα and ERβ, melatonin 1B receptor, chromodomain helicase DNA-binding protein 7 (CHD7), tryptophan hydroxylase 1, collagen type 1 α, interleukin-6, matrix metalloproteinase-3 and γ1-syntrophin (SNTG1) [34]. Genes like insulin-like growth factor IGF-1, estrogen receptors ERα and matrillin-1 were reported to be associated with curve severity [76-78]. The reasons for the different age of scoliosis onset still remains one of the questions to be answered. Whether differences in expression of VDR dependent genes Tob2 and Med13 in paravertebral muscles of the curve concavity observed between Adolescent and Juvenile Idiopathic Scoliosis group in this study are primary or secondary to the time of the scoliosis evolution merits further investigation.

## Conclusions

Alternative splicing of VDR mRNA occurs in paravertebral muscles and blood tissue of idiopathic scoliosis patients regardless the age of onset.

In Idiopathic Scolioses transcriptional activity and alternative splicing of VDR mRNA in osseous, cartilaginous, and paravertebral muscular tissues are tissue specific and equal on both sides of the curve.

The number of mRNA copies of VDRl izoform in paravertebral muscles of the curve concavity might be one of the factors differentiating Juvenile and Adolescent type of Idiopathic Scoliosis.

In paravertebral muscles, out of the 75 VDR responsive genes, Tob2 and Med13 genes differentiate Adolescent and Juvenile type of Idiopathic Scoliosis.

## Abbreviations

AIS: Adolescent Idiopathic Scoliosis; BMD: Bone mineral density; BMP: Bone morphogenetic protein; Caf1: Chromatin assembly factor-1; cAMP: Cyclic adenosine monophosphate; CDK8: Cyclin dependent kinase 8; DNA: Deoxyribonucleic acid; ERK1, ERK2: Extracellular-signal-regulated kinases 1, 2; Fi: Flexibility index; JIS: Juvenile Idiopathic Scoliosis; MAPK: Mitogen-activated protein kinase; MED13: Mediator complex subunit 13; mRNA: Messenger ribonucleic acid; QRT PCR: Quantitative Real Time Reverse Transcriptase Chain Reaction; RANKL: Receptor activator of nuclear factor kappa-B ligand; RAsag: Rotation angle relative to sagittal plane; RHi: Rib hump index; TGFβ: Transforming growth factor beta; Tob2: Transducer of erB-2; VDR: Vitamin D receptor; VDRl: Vitamin D receptor long isoform; VDRs: Vitamin D receptor short isoform.

## Competing interests

The authors declare that they have no financial or non-financial competing interests.

## Authors' contributions

RN participated in the design of the study, performed spinal surgeries, prepared tissue samples, performed radiological measurements and statistical analysis and drafted the manuscript. JS carried out molecular studies and participated in the design of the study. UM has been involved in the design of the study, analysis and interpretation of the data and helped to draft the manuscript. All authors read and approved the final manuscript.

## Pre-publication history

The pre-publication history for this paper can be accessed here:

http://www.biomedcentral.com/1471-2474/13/259/prepub
